# Neural Locus of Color Afterimages

**Published:** 2012-01

**Authors:** Qasim Zaidi

**Affiliations:** Graduate Center for Vision Research, SUNY College of Optometry, New York, USA

After fixating on a colored pattern, observers see a similar pattern in complementary colors when the stimulus is removed. Afterimages were important in disproving the theory that visual rays emanate from the eye, in demonstrating interocular interactions, and in revealing the independence of binocular vision from eye movements. Afterimages also proved invaluable in exploring selective attention, filling-in and consciousness. Proposed physiological mechanisms for color afterimages range from bleaching of cone photopigments to cortical adaptation, but direct neural measurements have not been reported.

We introduce a time-varying method for evoking afterimages, which provides precise measurements of adaptation and a direct link between visual percepts and neural responses. We then use *in vivo* electrophysiological recordings to show that all three classes of primate retinal ganglion cells exhibit subtractive adaptation to prolonged stimuli, with much slower time constants than those expected from photoreceptors. At the cessation of the stimulus, ganglion cells generate rebound responses that can provide afterimage signals for later neurons (Fig. 1). Our results indicate that afterimage signals are generated in the retina, but may be modified, like other retinal signals, by cortical processes. Therefore, evidence presented for cortical generation of color afterimages is explainable by spatiotemporal factors that modify all signals.

## Figures and Tables

**Figure 1.**
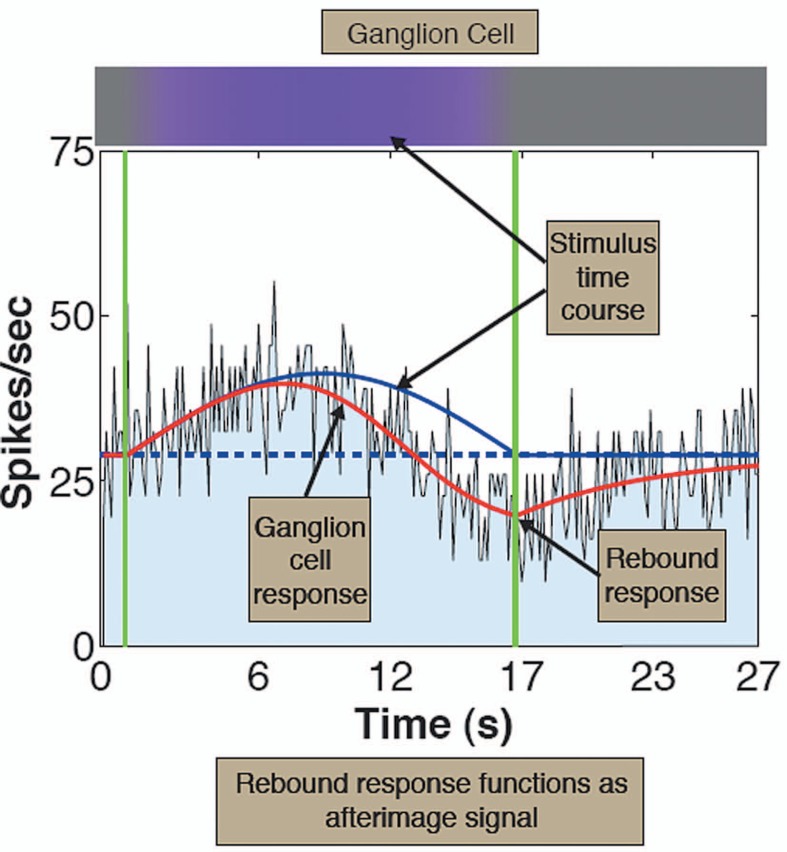
Ganglion cell responses to slow sinusoidal modulations: Histogram of +S-center KC spike responses to modulation towards Violet pole of Delta(S) axis. Solid red line: best fit of adaptation model. Solid blue line: response of cell without adaptation, which tracks the stimulus time-course. Dashed blue line: pre-stimulus response. Vertical green line: end of sinusoidal stimulus modulation.

